# *Agrobacterium rhizogenes*-mediated stable and transient transformation systems enable rapid functional validation of C6 aldehyde synthesis genes in peach

**DOI:** 10.1186/s12870-026-09136-y

**Published:** 2026-06-05

**Authors:** Yike Su, Mengtao Li, Xubin Wu, Bo Zhang, Xiangmei Cao

**Affiliations:** 1 Laboratory of Fruit Quality Biology/Zhejiang Key Laboratory of Horticultural Crop Quality Improvement, Zhejiang University, Zijingang Campus, Hangzhou, 310058 China; 2 State Key Laboratory for Development and Utilization of Forest Food Resources, Zhejiang A&F University, Hangzhou, 311300 China

**Keywords:** Fruit tree, Transgenic, Aroma, Lipoxygenase, Hydroperoxide lyase

## Abstract

**Background:**

The established genetic transformation system is essential for gene function research and genetic improvement of horticultural crops. The lack of efficient genetic transformation systems has long been a significant bottleneck for perennial fruit trees such as peach.

**Results:**

In this study, we provided an *Agrobacterium rhizogenes*-mediated transformation system by using stem segments of the ‘Hanlumi’ cultivar (*Prunus persica* L. Batsch). Through the optimization of explants, selection of the appropriate bacterial strain (MSU440), and determination of the infection concentration (OD_600_ = 1.0), we obtained transgenic callus and hairy roots with stable GFP expression. Additionally, a rapid transient transformation system was also developed using callus derived from stem and fruit tissues. Both systems were applied to functionally characterize genes involved in the biosynthesis of flavor-related C6 aldehydes. Overexpression of *PpLOX1* or *PpHPL1* in transgenic callus led to a significant increase in the levels of hexanal and *E*-2-hexenal, whereas RNAi silencing of *PpLOX6* or *PpHPL1* in callus resulted in a marked decrease. These results demonstrate that *PpLOX1*, *PpLOX6*, and *PpHPL1* are key genes in the peach C6 aldehyde pathway.

**Conclusions:**

The established transformation systems provide effective means for gene function validation and molecular breeding in peach, which have significant implications for the improvement of flavor quality.

**Supplementary Information:**

The online version contains supplementary material available at 10.1186/s12870-026-09136-y.

## Introduction

The research on gene function and genetic improvement of horticultural crops requires a genetic transformation system. In comparison with crops and model plants such as tomato, the genetic transformation system of perennial fruit trees remains imperfect [1]. For economic valuable fruit crop such as peach, a genetic transformation system with general applicability has not yet been established, which significantly restricts the development of peach biology research, molecular breeding and genetic engineering-assisted breeding. Stable genetic transformation systems have been developed for apple and strawberry in the Rosaceae family [2–5]. However, only a limited number of studies reporting successful peach transgene have been documented [4, 6], . Peach explants exhibit strong recalcitrance in vitro tissue culture, leading to achieve somatic embryo regeneration and organ regeneration. This is the primary factor influencing the establishment of peach genetic transformation systems [7–9].

Agrobacterium-mediated transformation is currently the mainstream genetic transformation means, commonly using *Agrobacterium tumefaciens* or *Agrobacterium rhizogenes*, of which *A. tumefaciens* has been widely used in model plants such as Arabidopsis and tomato, grain crops such as rice and maize [1], as well as horticultural plants such as apple and strawberry [5]. The key step in this process is the regeneration of buds and roots, which is difficult to achieve in many fruit trees. Compared with *A.tumefaciens*, *A. rhizogenes*-mediated genetic transformation has the advantages of wide applicability, high transformation efficiency, simple operation and short acquisition period, and has been gradually used in fruit trees in recent years, including litchi [10], citrus [11], apple [12], kiwifruit [1] and strawberry [13]. In citrus, CRISPR gene editing was achieved using *A. rhizogenes*, to obtain transgenic roots within 2 months and induced roots regeneration to obtain complete transgenic plants in 6 months, whereas *A. tumefacciens* required 12–18 months [11, 14–15]. In apple, the infection of *A. rhizogenes* was used to obtain transgenic hairy roots within 3 weeks with a conversion efficiency of 78.8% [1]. Currently, there are few studies on genetic transformation of peach using *A. rhizogenes*. Xu et al. [16] established a hairy root transgenic system by infecting hypocotyls, leaves, and shoot explants with *A. rhizogenes*, and demonstrated that PpMYB10.1 can activate the expression of downstream target genes to promote anthocyanin synthesis. Wu et al. [17] utilized *A. rhizogenes* to infect peach seedlings, demonstrating the regulatory role of the PpLBD16 transcription factor in lateral root primordia development.

For perennial fruit trees which obtaining stable transgenic plants is challenging, genetic transformation in callus tissues can simplify operational procedures, shorten research cycles, enhance expression stability, and more efficiently achieve gene function verification and functional gene screening. The difficulty of inducing callus varies greatly across different varieties and tissue types, with fruit callus induction being more challenging than that of seed embryo, leaf and stem segment [18]. Among Rosaceae plants, apple [19–24] and pear [25–30] fruit callus are widely used for functional validation of genes related to fruit quality, such as sugar and acid metabolism and anthocyanin synthesis. However, peach callus has been less utilized in gene function studies. Wang et al. [18] employed ‘ZaoJiubao’ peach anther callus to validate the functions of carotenoid and anthocyanin synthesis genes [18]. Inducing peach fruit callus and establishing efficient genetic transformation system have great significance for molecular breeding and quality improvement of peach.

Fruit flavor quality determines consumer preferences, with aroma quality being a crucial component that directly influences both flavor and overall sensory experience. C6 aldehyde volatile organic compounds (VOCs) such as hexanal and *E*-2-hexenal impart the ‘greeny-note’ and freshness characteristic of fruits. These VOCs also serve as precursors for the synthesis of esters with “fruity-note” [31]. Moreover, they are abundantly present in leaves and stems, participating in plant defense response. The synthesis of C6 aldehydes originate from the lipoxygenase (LOX) pathway, where unsaturated fatty acids undergo LOX oxidation to form hydroperoxy fatty acids, which are subsequently catalyzed by hydroperoxide lyase (HPL) to produce C6 aldehydes [32]. However, due to the lack of efficient and stable genetic transformation system, the key genes responsible for C6 aldehyde synthesis in peach have not been clearly identified.

This study established a stable *A. rhizogenes*-mediated genetic transformation system for peach through a comprehensive comparison of various explants, bacterial strains, and inoculation concentrations. Subsequently, this system was applied to the functional verification of genes associated with the synthesis of volatile organic compounds (VOCs). Additionally, an alternative homologous verification system was developed by transient expression in stem or fruit callus. Furthermore, the established stable and transient transformation systems effectively confirmed the roles of *PpLOX1* (Prupe.4G047800), *PpLOX6* (Prupe.2G005300), and *PpHPL1* (Prupe.3G213800) in the biosynthesis of “greeny-note” aldehydes in peaches.

## Materials and methods

### Plant materials

**Fig. 1:**
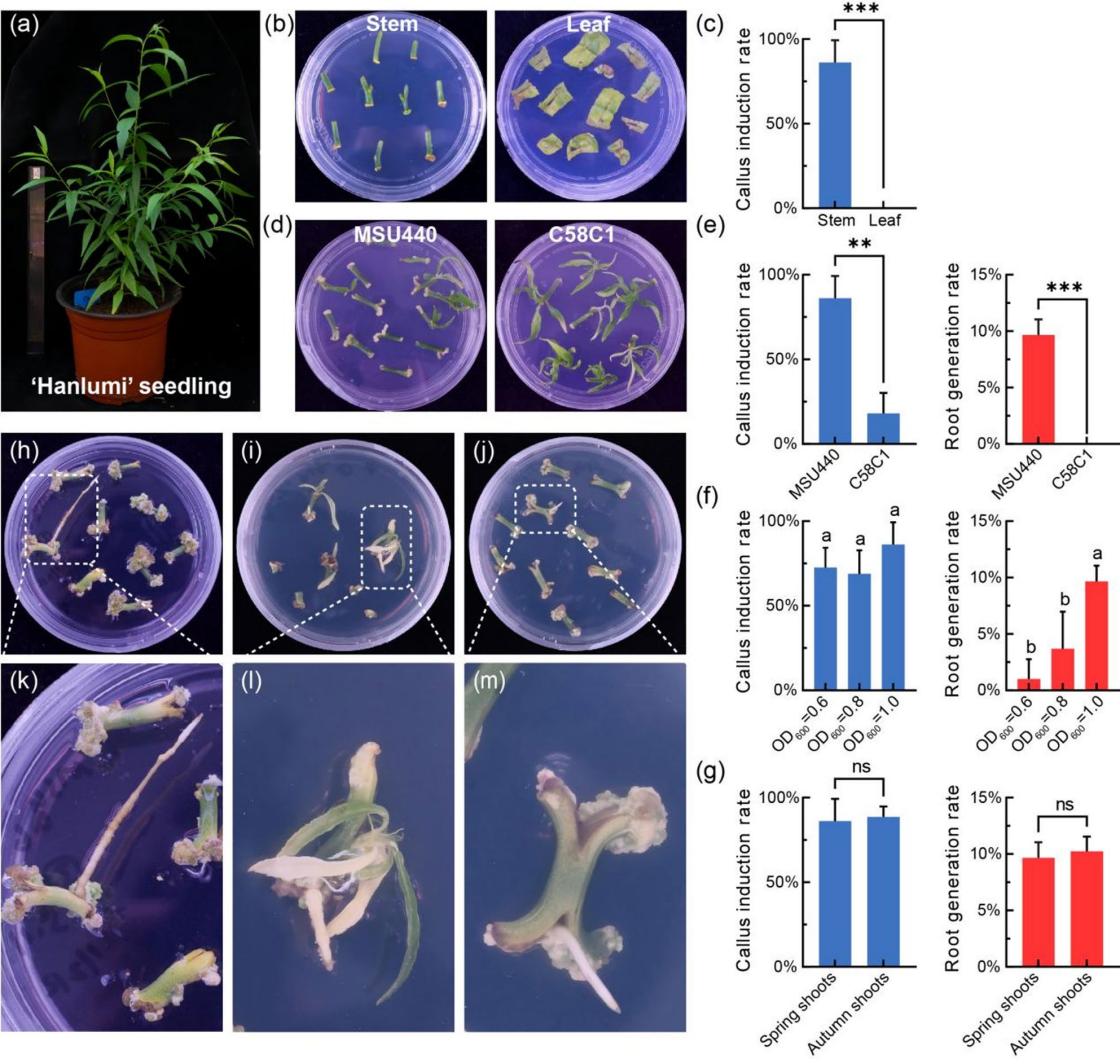
Establishment and optimization of *A. rhizogenes*-mediated genetic transformation system. (**a**), ‘Hanlumi’ peach seedling (4 months after sowing); (**b**), Infection of stem segments and leaf explants by *A. rhizogenes*, respectively (4 weeks after infection); (**c**), The callus induction rate of stem segments and leaf (4 weeks after infection). Statistical significance was determined using Student’s *t*-test; (**d**), Infection of stem segments by MSU440 and C58C1 strains (4 weeks after infection); (**e**), The callus induction rate and root generation rate of stem segments infected by MSU440 and C58C1 (6 weeks after infection). Statistical significance was determined using Student’s *t*-test; (**f**), The callus induction rate and root generation rate of stem segments infected by MSU440 at different concentrations. Different letters indicate significant differences between different groups (*P* < 0.05, one-way ANOVA, Tukey’s HSD post hoc test); (**g**), The callus induction rate and root generation rate of stem segments from spring and autumn shoots respectively infected by MSU440; (**h**-**j**), The callus and hairy root produced from stem segments infected by MSU440 carrying the GFP expression vector; (**k**-**m**), Partial zoom of the (**h**-**j**) plots, respectively. Statistical significance was determined using Student’s *t*-test.

The ‘Hanlumi’ (HLM) seedlings (*Prunus persica* L. Batsch) were cultivated in an artificial climate chamber under a 16/8 h light/dark cycle at 25 °C. After four months of growth, the leaves and stems of the seedlings were used as explants for establishment of *A. rhizogenes*-mediated genetic transformation system (Fig. 1a). The stems of ‘HLM’ seedlings, ‘HLM’ fruit, and ‘Hujingmilu’ (HJML) fruit 70 days after bloom were used for callus induction. Seeds and fruit materials of various peach varieties were obtained from the Melting Peach Research Institute of Fenghua, Zhejiang Province.

### Establishment and optimization of *A. rhizogenes*-mediated transgenic system

The *A. rhizogenes*-mediated transgenic system for hairy roots was adapted from the method described by Xu et al. [16] with appropriate modifications and optimizations. Details are described below. The medium components are listed in Table S1.

#### Preparation of *A. rhizogenes*

The pSAK277 expression vector containing the GFP reporter gene (the maps for the vector was shown in Figure S1) was transformed into *A. rhizogenes*, strains MSU440 and C58C1. The positive bacterial solution was added to liquid LB medium containing 50 mg L^− 1^ kanamycin (Kan), 50 mg L^− 1^ streptomycin (Strep), and 20 mg L^− 1^ acetosyringone (AS), and cultured overnight until OD_600_ = 1.0, centrifuged to collect the bacteria and resuspended with 1/2 MS (Murashige & Skoog Basal Salt Mixture, Phytotech) liquid medium containing 20 mg L^− 1^ AS to the appropriate concentration (the infection effects of OD_600_ = 0.6, 0.8 and 1.0 were compared).

#### Preparation of explants

Branches of ‘HLM’ seedlings were cut from the point of growth to the position of the three rounds of fully extended leaves, rinsed with water for 30 min and soaked in 75% alcohol for 1 min, washed twice in sterile water, and then soaked in 1% sodium hypochlorite for 10 min followed by five washes in sterile water and placed on sterile paper to absorb excess water. The leaves were separated, the main veins were removed, and the leaves were cut into slices of approximately 5 ~ 8 mm in length and width. After removal of the leaves, the stems were cut into 1 cm segments, and 2 mm length wounds were made perpendicular to the cross section of the stem segments.

#### Infection of the explants

The leaves and stem segments were immersed in *Agrobacterium* resuspension and infected on a shaker (28 ℃, 120 rpm) for 30 min. After filtration through sterile gauze, the residual bacterial solution on the surface was absorbed with sterile filter paper, and then the explants were co-cultured on MS co-cultured medium containing 20 mg L^− 1^ AS and incubated at 25 ℃ for 3 d in the dark. The explants were transferred to MS screening medium containing 400 mg L^− 1^ cefotaxime sodium (Cef) and 50 mg L^− 1^ Kan for screening culture at 25 ℃ under 16 h photoperiod conditions, and subcultured once every two weeks. The callus was produced after 4 w of infection and the root regenerated after about 6 w of infection.

#### GFP fluorescence and PCR detection of positive callus and regenerated roots

The callus and regenerated roots obtained after 6 w of infection were irradiated with GFP fluorescence excitation source (LUYOR-3415, LUYOR) for fluorescence detection, and the callus induction rate and root generation rate were measured. The callus and roots with green fluorescence were then frozen in liquid nitrogen for sampling, and genomic DNA and total RNA were extracted to detect gene insertion at the DNA level by PCR amplification (primers listed in Table S2) and expression at the transcriptional level by RT-qPCR (primers listed in Table S3), respectively.

#### The subculture of the transgenic callus

The positive callus after 6 w of infection were cut off and transferred to solid SH (Schenk & Hildebrandt Modified Basal medium, Phytotech) subculture medium Ⅰ containing 400 mg L^− 1^ Cef and cultured at 25 ℃ under dark conditions for 4 w (2 w subcultured once), after which the callus was transferred to SH subculture medium Ⅱ without antibiotics for successive subcultures.

### *A. rhizogenes*-mediated *PpLOX1/6* and *PpHPL1* transgenes

The full-length CDS sequences of *PpLOX1/6* and *PpHPL1* were constructed in pSAK277-GFP vector with the primers in Table S2, the recombinant vectors were transferred into MSU440, respectively. The transgenic system established above was used to carry out *PpLOX1/6* and *PpHPL1* transgene and obtain positive callus and hairy root. The empty pSAK277-GFP vector was used as control.

### ‘HLM’ stem callus induction

The stem segments from ‘HLM’ seedlings were used as explants to induce the callus according to the method of Pérez-Jiménez et al. [33] with some modification. Stems were sterilized according to the above procedure of explants preparation. Then the sterilized stem segments were placed on the MS induction medium Ⅰ containing 1.5 mg L^− 1^ 6-benzylaminopurine (BAP) and 0.5 mg L^− 1^ indole-3-butyric acid (IBA) as plant growth regulators, and induced under light until the production of callus (about 20 d), transferred to new MS induction medium successive cultured for 20 d, then the callus were cut off and transferred to SH subculture medium Ⅱ and successive subcultured at 25 ℃ under dark condition at 20 d intervals. The medium components are listed in Table S1.

### ‘HLM’ and ‘HJML’ fruit callus induction

Fresh fruit was immersed in 75% ethanol for 1 min, then rinsed three times with sterile water. Subsequently, it was soaked in a 2% NaClO solution for 13 min, followed by repeated rinsing with sterile water 5 ~ 6 times. After sterilization, place the fruit on sterile filter paper to absorb surface moisture. Remove the peel tissue, cut the pulp into 5 mm * 5 mm pieces, and inoculate them onto MS induction medium. To investigate the effect of light exposure on fruit callus formation, we established three light treatment groups: group A, induced for 20 d under continuous darkness; group B, induced for 10 d under darkness followed by transfer to light exposure (16 h light, 8 h dark) for 10 d; group C, induced for 20 d under continuous light exposure. We investigated the effects of three different media on callus induction from fruit, including MS, SH and WPM (Woody Plant Medium) induction medium (Table S1). After 20 ~ 30 d of induction, healthy callus tissue was excised and transferred to SH subculture medium Ⅱ for cultivation, with subculturing performed every 20 days. The medium components are listed in Table S1.

### Transient overexpression of *PpMYB10.1*, *PpLOX1* and *PpHPL1* in callus mediated by *A. rhizogenes*

The method of gene transient overexpression in callus was referred to Bai et al. [25] and Ni et al. [34] with some modification. The complete coding sequence (CDS) of *PpMYB10.1* (Prupe.3G163100), *PpLOX1* and *PpHPL1* were cloned into the pSAK277 vector with primers in Table S2. The recombinant vectors were transformed into *A. rhizogenes* MSU440, along with the empty vector control. The peach callus was induced from the stem of ‘HLM’ and the flesh of ‘HJML’. The transient transformation was conducted as follows: The bacterium was resuspended in SH liquid medium (OD_600_ = 0.8), the callus was carefully transferred into the bacterial suspension to soak for 10 min. The mixture was then filtered through three layers of cheesecloth, and the residual bacterial suspension on the surface was absorbed with sterile filter paper. The infected callus was transferred to SH subculture medium Ⅲ containing 20 mg L^− 1^ AS. After 3 days of coculture incubation in the dark (24 ℃, RH 60%), the infected callus was transferred to SH screening medium containing 400 mg L^− 1^ Cef and 50 mg L^− 1^ Kan for screening. Cultures were maintained in the dark for 4 days and then sampled. The medium components are listed in Table S1.

### Transient RNAi silencing of *PpLOX6* and *PpHPL1* in callus mediated by *A. rhizogenes*

A 400 bp specific sequence fragment of the coding regions of *PpLOX6* and *PpHPL1* was constructed into the pHELLSGATE2 gene silencing vector (the maps for the vector was shown in Figure S1) using the Gateway technology with the primers in Table S2. The recombinant vectors and the empty vector were transferred into *A. rhizogenes* strain MSU440. The callus was infected according to the transient overexpression method described above, and after 1 w of infection, the callus was subjected to RT-qPCR and phenotypic analysis.

### Volatile analysis by GC-MS

The analysis of fruit tissues-derived callus volatile compounds was conducted following the previously described method [35]. Weigh 1 g of the sample and transfer it into a 10 mL headspace vial. Add 1 mL of 20% CaCl_2_, followed by 1 mL of 200 mM ethylenediaminetetraacetic acid (EDTA). Introduce 10 µL of 2-octanol (0.8 mg mL^− 1^) as the internal standard. Secure the vial tightly then vigorously vortex the mixture. Subsequently, place the vial in the automated sample injector (Combi PAL, CTC Analytics, Agilent Technologies, USA) for SPME analysis. Identification of aromatic compounds relied on the NIST8 mass spectral library (NIST/EPA/NIH, USA) and reference standards. The quantitative analysis of aromatic compounds was based on peak areas generated using the internal standard.

### Gene expression analysis

The RNAprep Pure Plant Plus Kit (Tiangen) was use*d* to extract total RNA from the samples. The cDNA was synthesized using the HiScript II 1st Strand cDNA Synthesis Kit (Vazyme). For RT-qPCR analysis, a reaction volume of 20 µL was prepared, comprising 1 µL of forward and reverse primers (10 µM each), 6 µL of H_2_O, 2 µL of cDNA, and 10 µL of 2×SYBR (ChamQ Universal SYBR qPCR Master Mix, Vazyme). The internal control gene *PpTEF2* was used as a reference for RT-qPCR, and the primers were listed in Table S3.

### Phylogenetic tree construction

All LOX and HPL members in peach genome were screened out based on the genome annotation (https://phytozome-next.jgi.doe.gov/info/Ppersica_v2_1) and sequence similarity to members which have been identified in other species. Multiple sequence alignments of the amino acid sequences were performed using Clustal W with default parameters. The phylogenetic tree was constructed by neighbor-joining methods with 1,000 bootstrap replications by MEGA7.0, with the matrix of the evolutionary distances calculated by Poisson correction for the multiple substitutions. The genebank ID of members from other species are listed in Table S4.

### Subcellular localization analysis

Full length CDS of target genes were cloned into 35 S-e*GFP* vector with the primers in Table S2. The recombinant vectors were subsequently introduced into *A. tumefaciens* GV3101. To facilitate infiltration, the bacterial strains carrying the target gene were resuspended in an infiltration buffer (10 mM MgCl_2_, 10 mM MES, 150 µM AS, pH = 5.6) until reaching OD_600_ = 0.8. The vector was infiltrated into the leaves of 4-week-old *Nicotiana benthaminana* plants. Following a 48 h incubation period, the leaves were detached and subjected to observation using a confocal laser scanning microscope (LSM 780, Carl Zeiss, Oberkochen, Germany).

### Enzymatic activity of PpHPL1 protein *in vitro*

Heterologous protein expression and purification methods following previous study [35]. The full-length ORFs of *PpHPL1* was cloned into the pETN-6*HN expression vector (Clontech, Mountain View, CA, USA) with an N-terminal His-tag (primers are listed in Table S2). The construct was transformed into *E. coli* BL21(DE3) pLysS (Promega, Madison, WI, USA) to express the recombinant protein. Recombinant protein was purified by His TALON gravity column (Clontech) following the manufacturer’s instructions. The reaction system of 1 mL consisted as follows: 200 µL of the recombinant protein (500 ng µL^− 1^), 100 µL of 4.4 mg mL^− 1^ lipoxygenase from soybean (Sigma-Aldrich), 100 µL of substrate linoleic acid or linolenic acid (100 mM), and 600 µL of Tris-HCl buffer (100 mM Tris, 2 mM dithiothreitol, pH = 6.9). The mixture was transferred into a 4 mL vial and sealed, followed by incubation at 30 °C for 1 h. The products were analyzed using SPME-GC/MS.

### Statistical analysis

The figures were generated using GraphPad Prism 9.0 (GraphPad Software, LLC., San Diego, CA, USA). To assess the significance between the two samples, an unpaired Student’s *t* test was performed. Additionally, for evaluating the significance level among multiple groups, the least significant difference (LSD) of one-way analysis of variance (ANOVA) was employed. Statistical analyses were conducted using SPSS 19.0 software (SPSS Inc., Chicago, IL). Statistical significance levels were denoted as *P < 0.05 and **P < 0.01.

## Results

### Explant selection, strain and bacterial concentration affected the efficiency of *A. rhizogenes*-mediated genetic transformation

Leaf and stem tissues from ‘Hanlumi’ seedlings (Fig. 1a) were selected as explants to establish a genetic transformation system mediated by *A. rhizogenes* (MSU440) carrying the GFP expression vector. After 4 weeks of infection, the callus was formed at the wound of stem segments, with a callus induction rate of 86%, and roots grew after 6 weeks of infection, whereas the wounds of leaves gradually lignified after infection, and could not be induced to form callus (Fig. 1b, c). Therefore, the stem segments were selected as the explant for the subsequent assay. To further optimize the genetic transformation system, the infection effects of different *A. rhizogenes* strains (MSU440 and C58C1), different concentrations of bacterial solution (OD_600_ = 0.6, 0.8 and 1.0, respectively) and stem segments from different seasons (spring and autumn shoots) as explants were compared. Statistics on the efficiency of callus induction and root generation showed that after 4 weeks of MSU440 infection, most stem segments formed the callus, with a callus induction rate of 86% and root generation rate of 10%, whereas the stem segments infected with C58C1 showed only 18% callus induction rate and no root generation (Fig. 1d, e). Therefore, MSU440 was preferred as a more suitable root-generating *Agrobacterium* strain for peach stem segments. When the bacterial concentrations at OD_600_ = 1.0, the root formation rate peaked at 9.7%, which was 10 and 3 times higher than that of 0.6 and 0.8, respectively, although the differences in callus induction rates were not significant (Fig. 1f). In addition, the callus induction rates of stem segments from spring and autumn shoot were close to each other at 86% and 89%, respectively, and the root generation rates of both were also very close (Fig. 1g). Thus, the seasonal limitation of explant collection was largely avoided. The above statistical data on callus induction rate and root generation rate were obtained from three independent batches of experiments. Each batch involved infecting approximately 100 explant fragments (10 explants per plate, for a total of 10 culture plates). After the above optimization, stems were selected as explants and infected by MSU440 *A. rhizogenes* carrying the target gene with OD_600_ = 1.0 to achieve genetic transformation. After several batches of experiments, callus formation was observed approximately 20 d after infection, and hairy root began to form 40 d after infection (Fig. 1h-m).

**Fig. 2:**
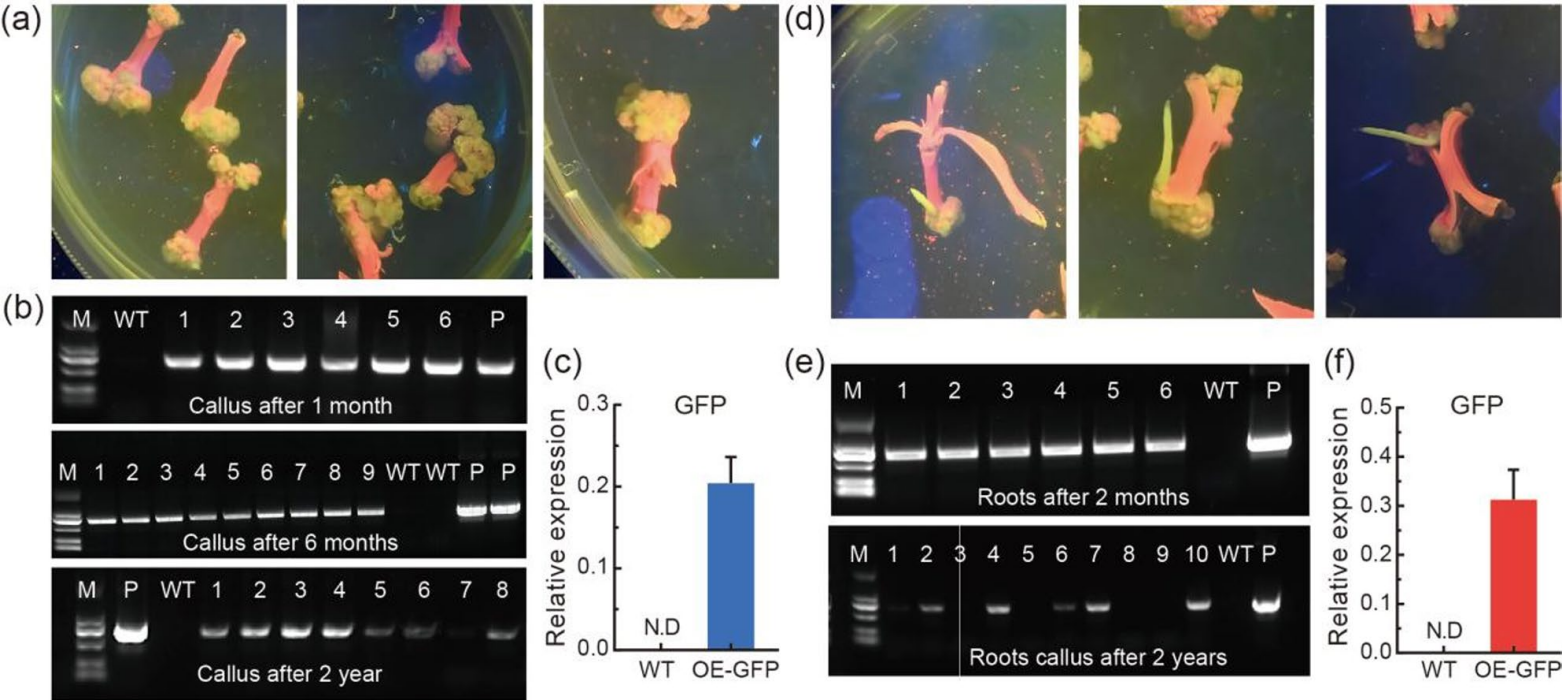
Transgene-positive detection of callus and roots obtained by *A. rhizogenes* infection. (**a**), The stem callus showed green fluorescence at the GFP fluorescence excitation wavelength; (**b**), PCR detection of the GFP coding sequence in stem callus continuously cultured for different times (1 month, 6 months and 2 years); M represents the marker, P represents the positive control with plasmid, WT represents the wild-type root and the numbers 1–9 represent the independent replicates of callus per plate; (**c**), The GFP gene was overexpressed in the stem callus (1 month); (**d**), The roots showed green fluorescence at the GFP fluorescence excitation wavelength (2 months); (**e**), PCR detection of the GFP coding sequence in the roots (2 months) and the callus induced from the roots (2 years); (**f**), The GFP gene was overexpressed in the roots (2 months).

To further verify the effect of genetic transformation, overexpression of the target gene *GFP* was detected. Under the irradiation of GFP-excited light, the callus formed at the wound of the stem one month after infection showed green fluorescence (Fig. 2a), indicating the overexpression of GFP. The transformed callus was also subjected to PCR, which showed that a 720 bp *GFP* gene band was cloned in all independent replicates of the transgenic callus, whereas there was no band in the control (WT) (Fig. 2b), and RT-qPCR also showed that *GFP* was highly expressed in the transgenic callus compared to the control (Fig. 2c). Furthermore, the *GFP* gene bands were still detected in the transgenic callus after 6 months and 2 years of continuous culture (Fig. 2b), indicating that the callus induced by infection with *A. rhizogenes* carrying the target genes could successfully integrate the target genes into the genome and achieve stable high expression.

Further investigation into gene transformation efficiency in root tissues, the results showed that the hairy root produced after 2 months of infection showed obvious green fluorescence (Fig. 2d), and PCR and RT-qPCR analyses also proved that the GFP gene was overexpressed in the hairy root (Fig. 2e, f). In addition, the transgenic roots were induced into callus and continued to be succeeded for two years, and the GFP gene bands could still be detected with a positive transgene rate of 71% (Fig. 2e). The above results demonstrated that this *A. rhizogenes*-mediated transgenic system could achieve sustained and stable high expression of the target genes in stem callus and root tissues.

### *A. rhizogenes*-mediated transformation system demonstrated that PpLOX1 and PpLOX6 are involved in the biosynthesis of volatile C6 aldehydes in peach

**Fig. 3:**
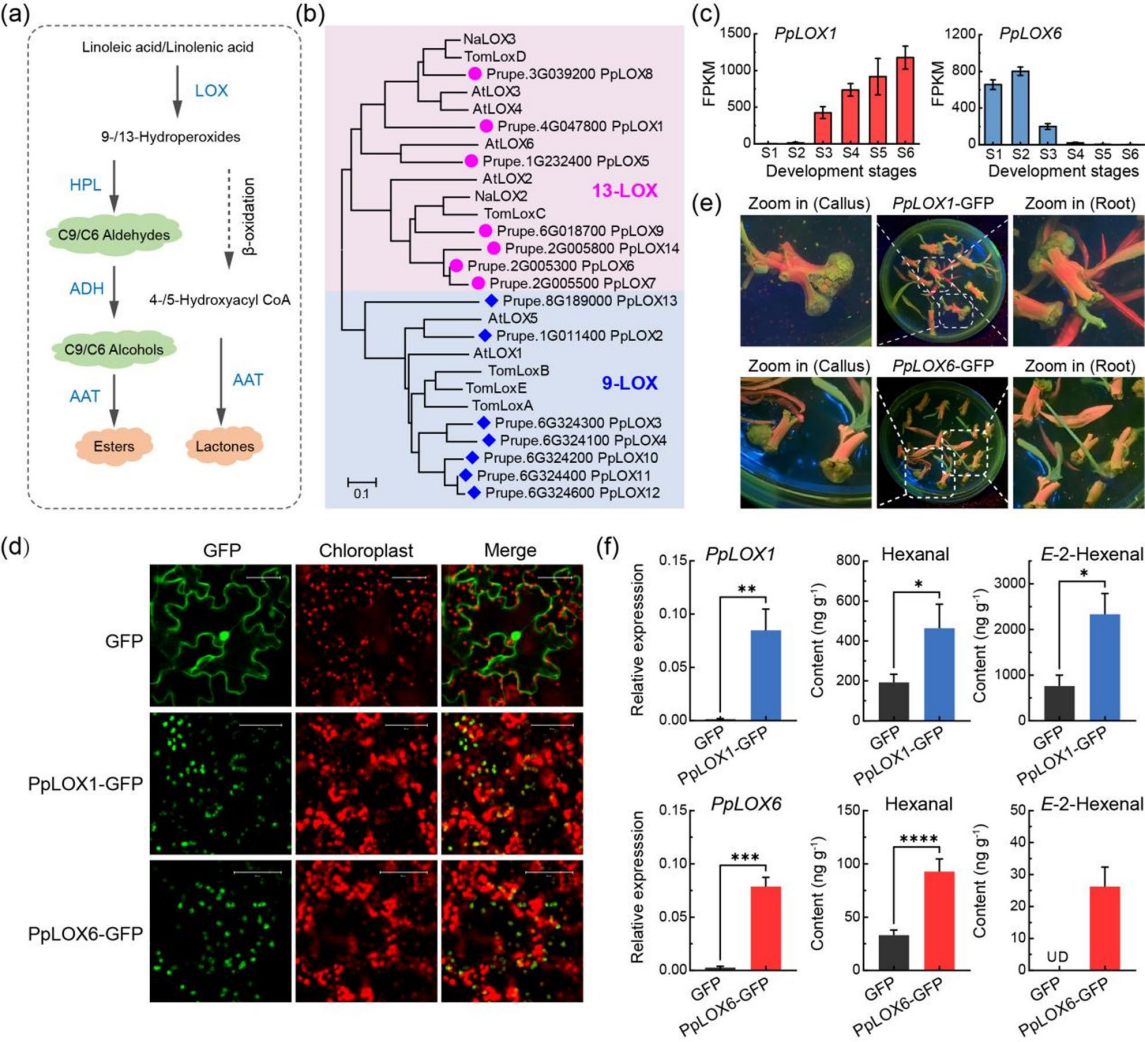
The *A. rhizogenes*-mediated transgenic system was used to demonstrate that PpLOX1 and PpLOX6 involved in the synthesis of C6 aldehydes in peach. (**a**), LOX metabolic pathway; LOX: lipoxygenase (LOX), HPL: hydroperoxide lyase, ADH: alcohol dehydrogenase, AAT: alcohol acetyltransferase; (**b**), Phylogenetic tree of LOX family members; (**c**), The transcript level of *PpLOX1* and *PpLOX6* during peach fruit ripening, S1-S6 refer to different developmental stages of peach fruit, which are 34, 50, 71, 108, 111, and 114 days after bloom, respectively; (**d**), Subcellular localization of PpLOX1 and PpLOX6 in tobacco leaf. Bar = 50 μm; (**e**), The stem callus and roots overexpressing PpLOX1 and PpLOX6 showed green fluorescence; (**f**), Overexpression of *PpLOX1* and *PpLOX6* in stem callus promote the synthesis of hexanal and *E*-2-hexenal; UD: Under the detection line. Statistical significance was determined using Student’s *t*-test.

C6 aldehyde volatiles, originating from fatty acids, are the main source of peach green odor, and serve as essential substrates for the synthesis of fruity esters, including hexanal, *E*-2-hexenal and *Z*-3-hexenal [31]. Lipoxygenase (LOX) is a key enzyme involved in the synthesis of C6 aldehydes, catalyzing the peroxidation of unsaturated fatty acids (Fig. 3a). Fourteen LOX family members were identified in peach, and phylogenetic analysis showed that seven were 13-LOX members and the others were 9-LOX members, of which PpLOX1 shares high similarity with tomato TomLOXD and tobacco NaLOX3, which have been identified as involved in jasmonic acid biosynthesis, and PpLOX6 is highly similar to tomato TomLOXC and tobacco NaLOX2, which have been proven to participate in VOCs synthesis (Fig. 3b). These two LOX members have the highest transcript abundance in peach fruit, and the transcript level of *PpLOX1* gradually increased during peach fruit ripening, whereas the transcript level of *PpLOX6* showed a decreased pattern (Fig. 3c, S1), and these two members were selected as candidates that may be involved in C6 aldehydes synthesis. Subcellular localization analysis showed that the both PpLOX1 and PpLOX6 exhibited a strong green fluorescence signal in chloroplasts, consistent with the chloroplast spontaneous fluorescence (Fig. 3d), indicating that PpLOX1 and PpLOX6 are localized in chloroplasts. The above transgenic system was used for their homologous validation in *vivo.* Stable transgenic callus and root tissues were obtained with *A. rhizogenes* carrying the target genes infiltrating the stem segment tissues of ‘HLM’ (Fig. 3e). RT-qPCR analysis confirmed the overexpression of *PpLOX1* and *PpLOX6* in the callus, while VOC profiles analysis demonstrated that the content of hexanal and *E*-2-hexenal in *PpLOX1* and *PpLOX6* transgenic callus tissues were significantly higher than in GFP control, with increases exceeding 2-fold (Fig. 3f). These results suggest that both PpLOX1 and PpLOX6 are involved in the synthesis of volatile C6 aldehydes in peach.

### *A. rhizogenes*-mediated transgenic system demonstrated that PpHPL1 catalyzes the biosynthesis of volatile C6 aldehydes in peach

**Fig. 4:**
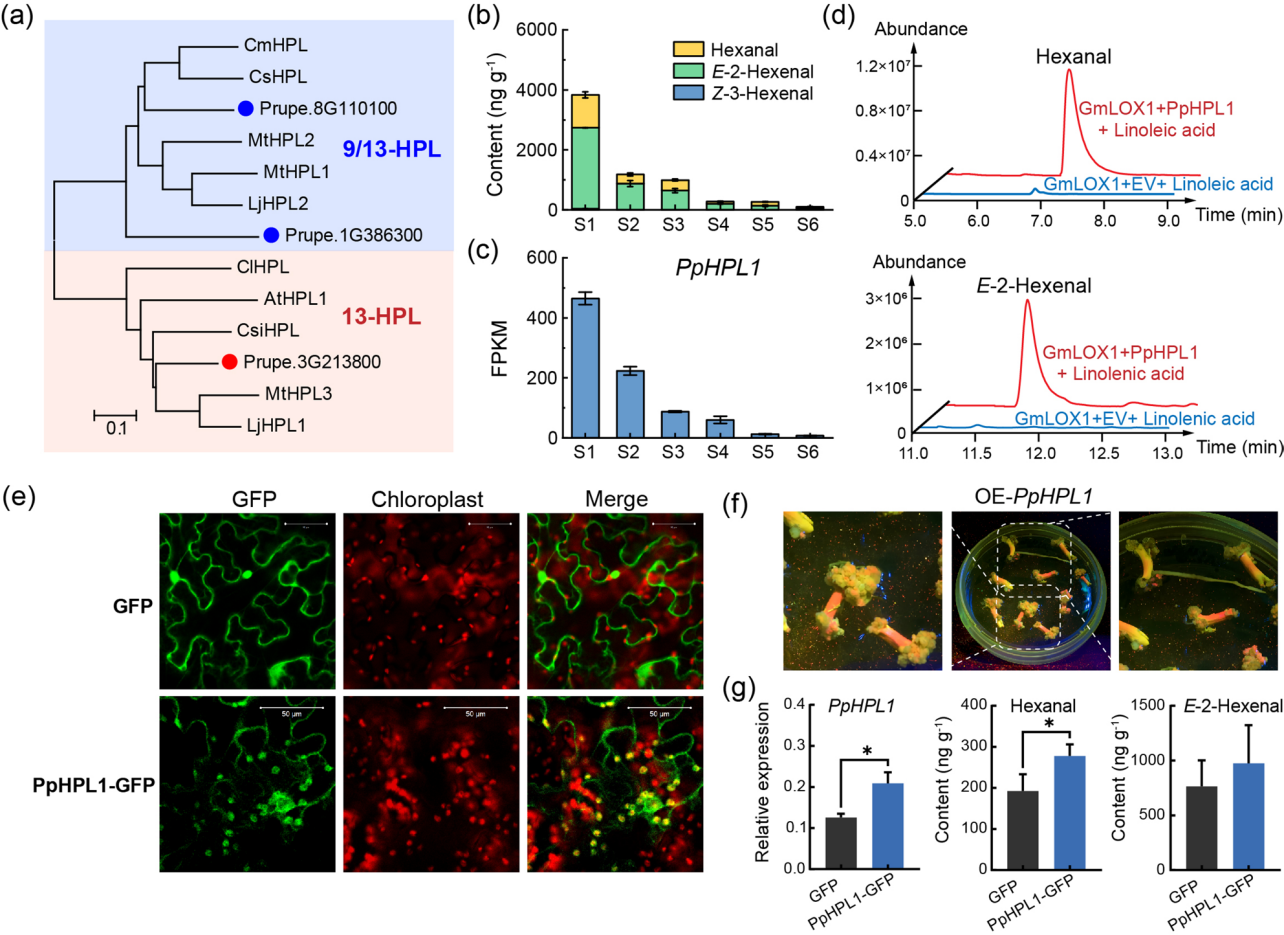
The *A. rhizogenes*-mediated transgenic system was used to demonstrate that PpHPL1 catalyzed the synthesis of C6 aldehydes in peach. (**a**), Phylogenetic tree of HPL family members; (**b**), The content of volatile C6 aldehydes during peach fruit ripening. S1-S6 refer to different developmental stages of peach fruit, which are 34, 50, 71, 108, 111, and 114 days after bloom, respectively; (**c**), The transcript level of *PpHPL1* during peach fruit ripening; (**d**), Enzymatic activity of recombinant PpHPL1 protein in vitro. (**e**), Subcellular localization of PpHPL1 in tobacco leaf. Bar = 50 μm; (**f**), The stem callus and root overexpressing PpHPL1 showed green fluorescence at the GFP fluorescence excitation wavelength; (**g**), Overexpression of PpHPL1 in stem callus promotes the synthesis of hexanal and *E*-2-hexenal. Statistical significance was determined using Student’s *t*-test

Previous studies have shown that hydroperoxide lyase (HPL) could directly catalyze the cleavage of 13-hydroperoxides to produce C6 aldehydes (Fig. 3a) [32], but the function of HPLs in peach has still not been systematically verified. Three HPL members were identified in peach, and phylogenetic analysis showed that PpHPL1 is a 13-HPL, similar to AtHPL1 in *Arabidopsis* (Fig. 4a), which has been confirmed to catalyze the synthesis of C6 aldehydes [36]. The transcript level of *PpHPL1* showed a continuous decrease pattern during fruit ripening, consistent with the change of C6 aldehydes content (Fig. 4b, c), while the other two members showing extremely low expression (Figure S2), so *PpHPL1* is considered a key candidate gene for catalyzing C6 aldehyde synthesis. Recombinant protein activity analysis was used to validate the function of PpHPL1 in vitro, and LOX enzyme from soybean (GmLOX1) was used to synthesize the precursors required for HPL. The results showed that GmLOX1 enzyme plus recombinant PpHPL1 protein could catalyze the synthesis of hexanal and *E*-2-hexenal with linoleic acid (18:2) and linolenic acid (18:3) as substrates, respectively (Fig. 4d), indicating that PpHPL1 could catalyze hydroperoxides cleavage to produce C6 aldehydes. Subcellular localization analysis showed that the PpHPL1 exhibited a strong green fluorescence signal in chloroplasts, consistent with the chloroplast spontaneous fluorescence. In addition, it also showed a certain fluorescent intensity in cytoplasm (Fig. 4e). The above transgenic system was used for homologous validation of *PpHPL1* in *vivo.* Stable transgenic callus and hairy root were obtained (Fig. 4f). RT-qPCR analysis confirmed the overexpression of *PpHPL1* in the callus after one month of culture, and VOCs analysis demonstrated a 44% increase in the content of hexanal compared to the GFP control (Fig. 4g), indicating that PpHPL1 could directly catalyze the synthesis of C6 aldehydes.

### Induction of callus using stem and fruit tissues as explants

**Fig. 5:**
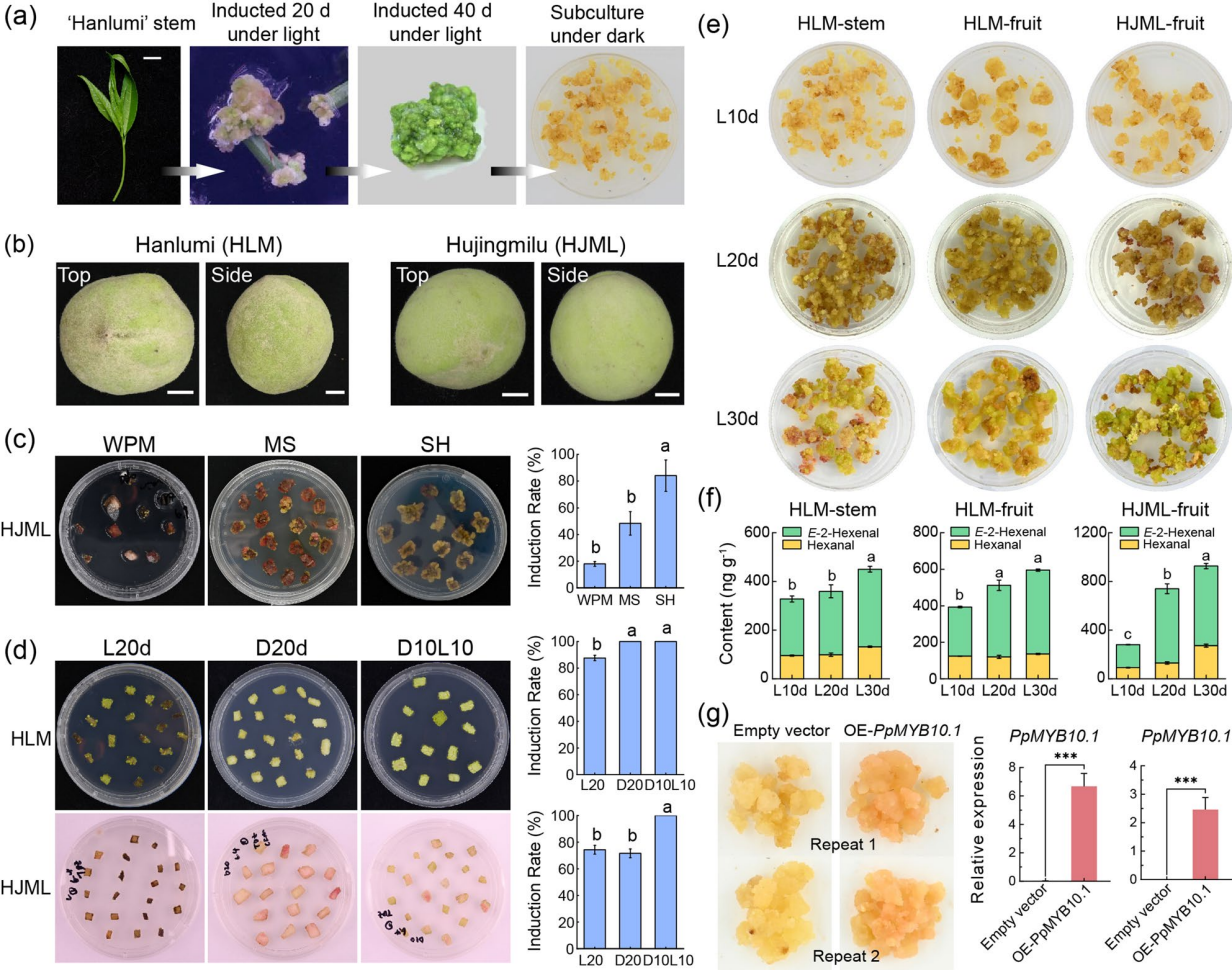
Optimization of the induction system of peach stem and fruit callus, and transient overexpression of *PpMYB10.1* in stem callus. (**a**), Induction and subculture of peach stem callus; (**b**), ‘Hanlumi’ and ‘Hujingmilu’ fruits used for callus induction; (**c**), Effect of different media on the induction rate of fruit callus. WPM represent Wood Plant medium, MS represent Murashige & Skoog medium and SH represent Schenk & Hildebrandt medium; (**d**), Effect of light condition on the induction rate of fruit callus; L20 represents 20 days under light, D20 represents 20 days under dark, and D10L10 represents 10 days under dark then 10 days under light; (**e**), Influence of light condition on the growth of callus during subculture; (**f**), Effect of light condition on the content of C6 aldehydes in callus; (**g**), Overexpression of *PpMYB10.1* in stem callus resulted in reddening of the callus. Statistical significance was determined using Student’s *t*-test.

Since the above stable transgenic system depends on the acquisition of explants, and the efficiency of obtaining transgenic root is low and the growth period is long, the transformation of callus tissue can realize the rapid verification of genes function. We used the young stem segments of ‘Hanlumi’ as explants to induce callus under light for 20 d, the wound could produce callus, and a large number of callus could be obtained after 40 d of induction. Subsequently, the callus was cut off and transferred to successively subculture under dark conditions, the green callus gradually turned to yellow, and the healthy growth state can be maintained by subculturing every 30 d (Fig. 5a).

Compared to stem callus, the induction of fruit callus is more difficult, and we compared the induction effects with different cultivars, different media and different light conditions to optimize the system. Peach cultivars ‘Hanlumi’ (HLM) and ‘Hujingmilu’ (HJML) fruits 70 d after bloom were selected as explants (Fig. 5b), and after sterilisation, the flesh tissue was cut into 5 mm × 5 mm pieces and inoculated into the callus induction medium. The suitable medium formulations were screened using ‘HJML’ fruits, and the results showed that SH medium is more suitable for fruit callus induction compared to WPM and MS medium. After 40 d of induction, the callus induction rate on SH medium was more than 80%, which was twice that on MS medium and three times that on WPM medium, and the callus growth on SH medium was healthier (Fig. 5c). So the SH medium was selected as fruit callus induction medium.

Light is an important environmental factor that regulates plant development and metabolism, and light conditions affect the efficiency of fruit callus induction. For ‘HLM’ fruits, proper dark conditions promote the formation of callus compared to prolonged exposure to light. The callus induction rate after 20 d under dark conditions (D20) and 10 d of dark induction followed by 10 d of light induction (D10L10) both reached 100% (Fig. 5d). While for ‘HJML’ fruits, proper light conditions (D10L10) promote the formation of callus compared to prolonged exposure to light or under dark condition (Fig. 5d). These results suggest that suitable conditions for fruit callus induction vary slightly between cultivars, and a certain amount of light could promote fruit callus formation, but prolonged light exposure would accelerate the callus browning and death. The appearance of some phenotypes requires a certain intensity and duration of light induction, such as photomorphogenesis, pigment synthesis, etc. Therefore, we further investigated the effect of light on the growth status of callus during continuous succession, and the results showed that short-term light exposure (L10d) had no significant effect on stem (‘HLM’) and fruit (‘HLM’ and ‘HJML’) callus, but continuous light exposure for more than 20 d induced chlorophyll accumulation and browning in callus (Fig. 5e), so it was unsuitable to subculture peach callus under long-term light conditions. Furthermore, light significantly induced the synthesis of volatile C6 aldehydes in callus (Fig. 5f), especially in ‘HJML’ fruit callus, which may be related to the chloroplast localization of PpLOX1/6 and PpHPL1, which catalyze C6 aldehydes synthesis (Figs. 3f and 4e). In addition, there are significant differences in the content and composition of VOCs in callus from different tissues or varieties (Figure S3), which means that appropriate callus materials should be selected when verifying the function of flavor-related genes.

To carry out homologous validation of gene function using the obtained callus, we used *A. rhizogenes* to infect ‘HLM’ stem callus to overexpress the transcription factor *PpMYB10.1*, which has been identified to activate the expression of genes involved in anthocyanin synthesis by interacting with PpbHLH3 and WD40 [37]. After 1 week of infection, callus overexpressing *PpMYB10.1* showed a light red colour compared to the empty vector control, and two independent experimental replicates showed similar phenotypes (Fig. 5g). RT-qPCR analysis confirmed that the transcript level of *PpMYB10.1* in the reddened callus increased 513-fold and 243-fold in two replicates, respectively (Fig. 5g). These results demonstrated that genetic transformation and homology verification of gene function could be achieved by infecting callus with *A. rhizogenes*.

### Transient overexpression and RNAi silencing in callus proved that *PpLOX1/6* and *PpHPL1* were involved in C6 aldehydes synthesis

**Fig. 6:**
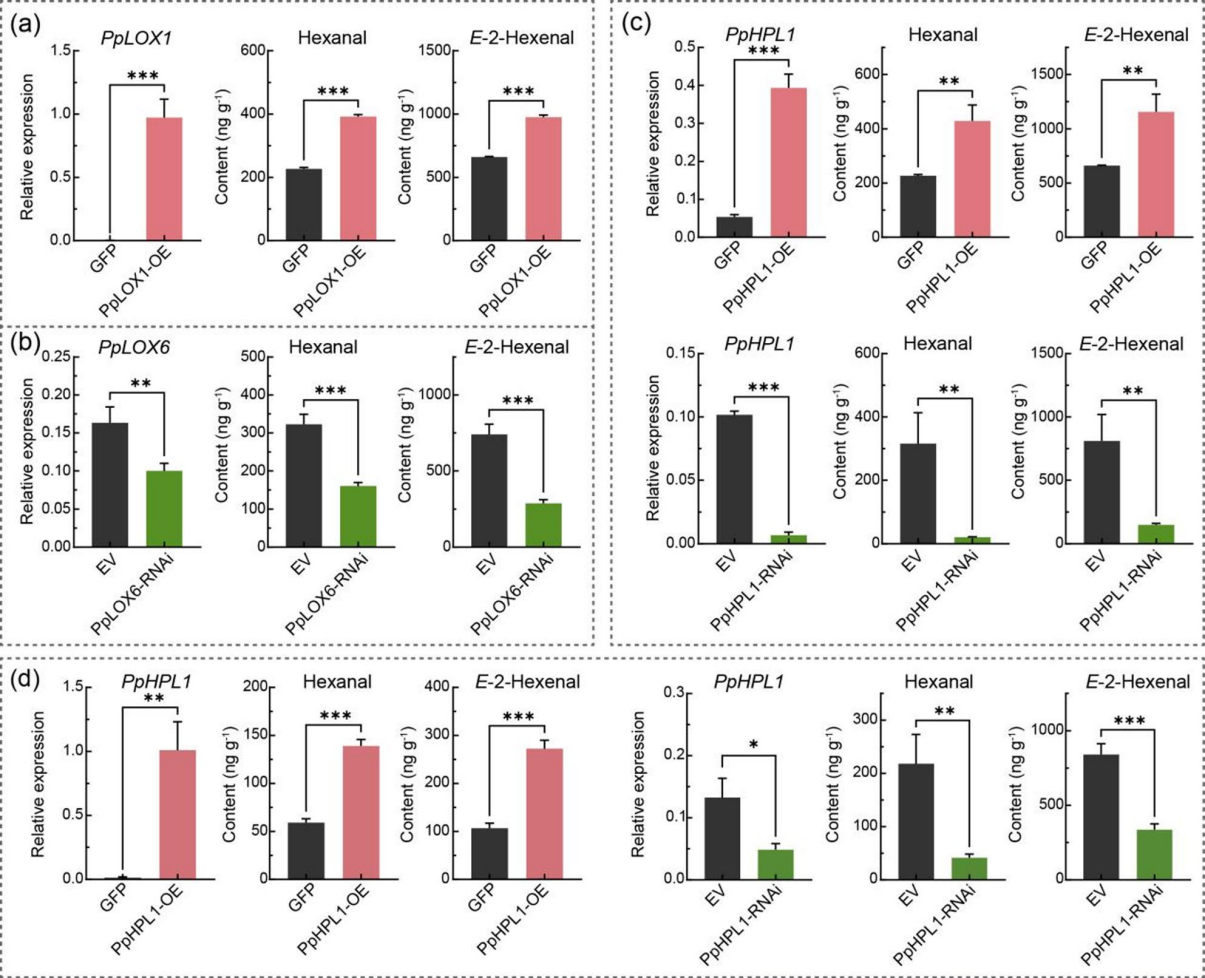
The transient overexpression system in callus was used to demonstrate that *PpHPL1*, *PpLOX1* and *PpLOX6* involved in the synthesis of volatile C6 aldehydes in peach. Statistical significance was determined using Student’s *t*-test. (**a**), Transient overexpression of *PpLOX1* in stem callus; (**b**), Transient RNAi-silence of *PpLOX6* in stem callus; (**c**), Transient overexpression and RNAi-silence of *PpHPL1* in stem callus; (**d**), Transient overexpression and RNAi-silence of *PpHPL1* in ‘HJML’ fruit callus.

The role of *PpLOX1/6* and *PpHPL1* in C6 aldehydes synthesis was further validated using the callus genetic transformation system. The overexpression of *GFP*, *PpHPL1-GFP* and *PpLOX1-GFP* in stem callus and fruit callus all showed significant green fluorescence (Figure S4). Compared with GFP control, the contents of hexanal and *E*-2-hexenal in *PpLOX1* overexpression callus increased by 73% and 48% respectively (Fig. 6a). As *PpLOX6* has a relatively high background expression level, RNAi silencing was carried out, and the results showed that silencing of *PpLOX6* was accompanied with a 50% decrease in hexanal and a 60% decrease in *E*-2-hexenal (Fig. 6b). The results demonstrated that both PpLOX1 and PpLOX6 are involved in the synthesis of hexanal and *E*-2-hexenal in peach.

*PpHPL1* overexpression and RNAi silencing were carried out on stem callus and the results showed that PpHPL1-GFP overexpression resulted in a 1.9-fold and 1.8-fold increase in hexanal and *E*-2-hexenal content, respectively; however, RNAi silencing drastically reduced the expression of *PpHPL1* in callus compared to the control (Fig. 6c), resulting in more than a 90% reduction of both hexanal and *E*-2-hexenal in callus. In addition, overexpression and RNAi silencing of *PpHPL1* in ‘HJML’ fruit callus also showed similar results to those in stem callus (Fig. 6d). The above results further confirmed the roles of *PpLOX1*, *PpLOX6* and *PpHPL1* in catalyzing the synthesis of C6 aldehydes aromatic compounds, and proved that the overexpression and RNAi silencing system based on callus developed in this study could be successfully applied to the functional verification of genes related to peach fruit flavor.

## Discussion

This study investigated factors affecting *A. rhizogenes* infection efficiency, including explant materials, bacterial strains, and bacterial suspension concentration, ultimately establishing a genetic transformation system for peach plants based on *A. rhizogenes*. Results demonstrated that using stem segments as explants, with MSU440 as transforming strain, and infecting at an OD_600_ = 1.0 bacterial suspension concentration yields more efficient transformation (Fig. 1). Both induced stem callus and hairy root successfully achieved transgenic expression. The infection efficacy of different strains shows significant variation. Compared to MSU440, C58C1 infection only induced minimal callus and failed to induce rooting (Fig. 1e). Xu et al. [16] also confirmed that MSU440 is a suitable *A. rhizogenes* strain for peach transformation. In previous study, explants for *A. rhizogenes* infection primarily derived from sterile seedlings [16], which required substantial seed consumption and extended cultivation cycles. This study directly utilized tender stem segments from cultivated peach plants, with no specific requirements for tree age, making the materials readily accessible. Following sterilization, these segments met the requirements for *A. rhizogenes* infection, eliminating reliance on tissue-cultured seedlings and significantly reducing labor, material, and time costs.

Unlike the results reported by Xu et al. [16], hairy roots were not successfully induced in infected leaves in this study, which may be affected by the genotype of peach. Xu et al. selected ‘Shantao’, ‘Shengli’ and ‘Lvhuajiuhao’, while we selected ‘hanlumi’ (Fig. 1). *A. rhizogenes* infection of peach stem explants induced extensive callus. Detection results revealed stable overexpression of the transgene in callus tissue, with transformation efficiency approaching 100%, and remaining detectable after 24 months of continuous subculture (Fig. 2d). Two months post-infection, transgenic hairy roots were successfully obtained, and sustained high expression of the introduced gene was observed in callus induced from these roots (Fig. 2e). This indicates that the *A. rhizogenes-*mediated transformation system established in this study enables stable overexpression of genes in both stem callus and hairy roots. However, the process of inducing bud formation from callus or hairy root in peach remains challenging. Studies indicate that pre-transfer of genes related to apical meristem development or stem cell activity significantly enhances transformation and regeneration efficiency, such as the *MdBBM1* [38] and *MdWOX* [1] in apple.

Since the above stable transgenic system relies on the explants acquisition, the efficiency of obtaining transgenic roots is low, and the growth period of positive roots is long, so it is very necessary to explore a more efficient and convenient genetic transformation system for rapid gene function validation. In recent years, the validation of peach fruit quality related gene function has predominantly relied on transient transformation of fruit tissues, using high-pressure permeation, immersion, and injection [39–44]. However, this process involves complex operations and is limited by the fruit’s growth season. Additionally, experimental stability and reproducibility are affected by the maturity of the fruit. The application of callus for homologous verification of gene functions can effectively address these challenges. Given the significant variations in metabolite profiles among callus tissues from different genetic backgrounds and tissue sources (Figure S3), targeted selection of appropriate callus materials is crucial for gene functional validation. The induction of fruit callus is more challenging than that of peach leaves, stems, and anthers [18], and its sausceptibility to browning leads to high mortality during subculturing. We systematically investigated the factors affecting fruit callus induction, such as culture medium formulation and light. The results showed that high potassium nitrate SH culture medium was more suitable for fruit callus induction, and the addition of PVP (1 g L^− 1^) in the culture medium could effectively inhibit callus browning; the influence of light on fruit callus induction showed variety differences. In general, moderate light can promote callus formation, but too long light will induce the synthesis of anthocyanins and phenols, and accelerate the browning and death of callus (Fig. 5e). The flesh of ‘HJML’ accumulates anthocyanins more readily under light, while ‘Hanlumi’ exhibits lower sensitivity to light. In addition, light conditions also influence the accumulation of VOCs in callus (Fig. 5f), making post-transformation cultivation conditions crucial for phenotype detection. in callus.

We selected the *PpMYB10.1* transcription factor which has been identified to regulate peach anthocyanin synthesis and *GFP* reporter gene, as target genes to establish transient expression systems in stem and fruit callus tissue [37]. During the experiment, *A.tumefaciens* was attempted for genetic transformation. However, GV3101 carrying *PpMYB10.1* showed minimal red coloration after infecting callus, while MSU440 carrying *PpMYB10.1* gene induced significant red coloration after infecting callus (Fig. 5g). Furthermore, MSU440 carrying the *GFP* gene exhibited strong green fluorescence after callus infection, indicating that *A. rhizogenes* may possess enhanced infectivity (Figure S4). Consequently, we established an *A. rhizogenes*-mediated transient callus transformation system.

To validate the reliability of the established stable genetic transformation system and callus transient transformation system for peach flavor quality research, this study conducted functional verification on three key candidate genes *PpLOX1*, *PpLOX6*, and *PpHPL1* which were screened as potential contributors to the synthesis of green peach aroma. Through *A. rhizogenes* infection of ‘HLM’ stem segments, stable transgenic callus tissues were obtained. VOCs profile analysis revealed significantly increased of hexanal and *E*-2-hexenal content in *PpLOX1*- and *PpLOX6*- overexpressed transgenic callus, while *PpHPL1*-overexpressed callus showed markedly elevated hexanal content (Figs. 3g and 4g). Furthermore, based on background expression levels of each gene in stem and fruit callus, we performed transient overexpression and suppression of *PpHPL1*, overexpression of *PpLOX1*, and suppression of *PpLOX6*, further confirming their catalytic roles in volatile hexanal and *E*-2-hexenal synthesis (Fig. 6). These results demonstrate that the two gene homology verification systems established in this study can be applied to functional analysis of genes related to peach flavor quality. The transient expression system utilizing stem and fruit callus enables both overexpression and RNAi silencing of genes, allowing rapid validation of gene functions and facilitating high-throughput screening. This transient genetic transformation system provides innovative methodologies and insights for plant research, particularly in fruit tree where genetic transformation systems are unestablished or challenging to establish. Although quantifying transient transformation efficiency remains difficult, the results demonstrate that overexpression of target genes significantly increases their expression levels alongside substantial elevations in related VOCs contents. Conversely, gene silencing leads to decreased expression levels of the target gene and a corresponding reduction in VOCs content (Fig. 6). Collectively, the positive and negative validation of multiple gene functions has proved the maturity, efficacy and stability of this system.

Although the hairy root system is a useful tool for genetic transformation and for elucidating the metabolic physiology of fruits, it has some limitations compared to whole-fruit systems. Analysis of the VOCs content in roots and root-derived callus reveals that these models cannot fully encompass all metabolites present in fruits or comprehensively reveal the metabolic network changes during ripening processes. Additionally, significant differences in VOC composition and content exist between different tissues and their corresponding callus (Figure S3), suggesting that these systems are suitable for validating conserved metabolic pathways across various tissues, or for selecting appropriate systems based on the tissue-specificity of metabolites. Researchon fruit-specific metabolism (such as aromatic esters and lactones) still relies on intact fruit systems or further exploration of callus derived from mature fruits.

## Conclusion

Peaches are an important fruit, and their flavor strongly influence consumer acceptance. However, the lack of efficient genetic transformation systems has hindered research on peach fruit flavor, forcing many gene functions to be validated in other species. This study provides two effective strategies for verifying gene function in peach. First, infecting peach stem segments with *A. rhizogenes* produces stable transgenic callus and hairy roots within 6 ~ 8 weeks, enabling rapid functional testing genes involved in metabolism and root development. Second, a transient transformation system using callus from stem and fruit tissues allow both overexpression and silencing of target genes, overcoming limitations of instability and tissue-dependency in traditional methods. Using these systems, we confirmed that *PpLOX1*, *PpLOX6* and *PpHPL1* promote the production of flavor-impacting C6 aldehydes volatiles. These transformation systems advance molecular breeding and improve flavor quality in peach.

## Supplementary Information

Supplementary Material 1: Table S1. The medium components used in this study. Table S2. Primers used for target genes cloning and vectors conduction. Table S3. Primers used for gene expression by RT-qPCR. Table S4. Gene IDs of the LOX and HPL members in the phylogenetic tree.

Supplementary Material 2: Figure S1. The sequence maps of pSAK277 and pHELLSGATE2 vectors. Figure S2. The gene expression pattern of *PpLOXs* and *PpHPLs* during peach fruit ripening. Figure S3. The composition and content of VOCs in different tissues (fruits, stems, roots) and the callus tissue derived from them. Figure S4. GFP fluorescence detection of callus with transient overexpression of *PpLOX1* and *PpHPL1* by *A. rhizogenes*.

Supplementary Material 3: The original versions of the images in the article.

## Data Availability

The datasets used and/or analysed during the current study are available from the corresponding author on reasonable request.
