# Supplementary material for: Agrobacterium rhizogenes-mediated stable and transient transformation systems enable rapid functional validation of C6 aldehyde synthesis genes in peach
Source: BMC Plant Biol. 2026 Jun 5;26:1355. doi: 10.1186/s12870-026-09136-y (PMC13459506; doi:10.1186/s12870-026-09136-y)
Supplement: Supplementary file 1 [file 12870_2026_9136_MOESM2_ESM.docx]

***Agrobacterium rhizogenes*-mediated stable and transient transformation systems enable rapid functional validation of C6 aldehyde synthesis genes in peach**

**Yike Su^1^, Mengtao Li^1^, Xubin Wu^1^, Bo Zhang^1^, Xiangmei Cao^1, 2^**

**Corresponding author: Xiangmei Cao. E-mail: [xmcao@zju.edu.cn](mailto:xmcao@zju.edu.cn)**

*^1^ Fruit Science Institute/Zhejiang Key Laboratory of Horticultural Crop Quality Improvement, Zhejiang University, Zijingang Campus, Hangzhou 310058, China.*

*^2^ State Key Laboratory for Development and Utilization of Forest Food Resources, Zhejiang A&F University, Hangzhou, 311300, China*

**Figure S1.** The sequence maps of pSAK277 and pHELLSGATE2 vectors.

**Figure S2.** The gene expression pattern of *PpLOXs* and *PpHPLs* during peach fruit ripening.

**Figure S3.** The composition and content of VOCs in different tissues (fruits, stems, roots) and the callus tissue derived from them.

**Figure S4.** GFP fluorescence detection of callus with transient overexpression of *PpLOX1* and *PpHPL1* by *A. rhizogenes*.
