# Supplementary material for: Agrobacterium rhizogenes-mediated stable and transient transformation systems enable rapid functional validation of C6 aldehyde synthesis genes in peach
Source: BMC Plant Biol. 2026 Jun 5;26:1355. doi: 10.1186/s12870-026-09136-y (PMC13459506; doi:10.1186/s12870-026-09136-y)
Supplement: Supplementary file 2 [file 12870_2026_9136_MOESM3_ESM.docx]

***Agrobacterium rhizogenes*-mediated stable and transient transformation systems enable rapid functional validation of C6 aldehyde synthesis genes in peach**

**Yike Su^1^, Mengtao Li^1^, Xubin Wu^1^, Bo Zhang^1^, Xiangmei Cao^1, 2^**

**Corresponding author: Xiangmei Cao. E-mail: [xmcao@zju.edu.cn](mailto:xmcao@zju.edu.cn)**

*^1^ Fruit Science Institute/Zhejiang Key Laboratory of Horticultural Crop Quality Improvement, Zhejiang University, Zijingang Campus, Hangzhou 310058, China.*

*^2^ State Key Laboratory for Development and Utilization of Forest Food Resources, Zhejiang A&F University, Hangzhou, 311300, China*

Figure 1 b. Infection of stem segments and leaf explants.

Figure 1 d. Infection of stem segments by MSU440 and C58C1 strains.

Figure 1 h, i, j. The callus and root hair produced from stem segments infected by MSU440.

Figure 2 a, d. The stem callus and roots showed green fluorescence at the GFP fluorescence excitation wavelength.

Figure 2 b

Figure 2 e

Figure 3 e. The stem callus and roots overexpressing *PpLOX1* and *PpLOX6* respectively showed green fluorescence.

Figure 4 f. The stem callus and root overexpressing *PpHPL1* showed green fluorescence.

Figure 5a: HLM stem, callus induced 20 d under light, and subculture under dark.

Figure 5c: WPM, MS and SH.

Figure 5d: HLM-L20, D20 and D10L10.

Figure 5d: HJML-L20, D20 and D10L10

Figure 5e: HLM-Stem-L10d, L20d, L30d

Figure 5e: HLM-Fruit-L10d, L20d, L30d

Figure 5e: HJML-Fruit-L10d, L20d, L30d

Figure 5g: Empty vector control and *PpMYB10.1*-OE.

Figure S4: GFP fluorescence detection of ‘HLM’ stem callus with transient overexpression of *PpLOX1* and *PpHPL1* by *A. rhizogenes*.

Figure S4: GFP fluorescence detection of ‘HJML’ fruit callus with transient overexpression of *PpHPL1* by *A. rhizogenes*.
